# Proteins lead the way: JAK inhibitors as a new treatment option in toxic epidermal necrolysis

**DOI:** 10.1038/s41392-024-02122-6

**Published:** 2025-02-03

**Authors:** Benjamin Klein, J. Michelle Kahlenberg, Johann E. Gudjonsson

**Affiliations:** 1 Department of Dermatology, Allergology and Venereology, Leipzig University Medical Center, University of Leipzig, Leipzig, Germany; 2 Department of Internal Medicine, Division of Rheumatology, University of Michigan, Ann Arbor, MI USA; 3 Department of Dermatology, University of Michigan, Ann Arbor, MI USA

**Keywords:** Inflammation, Inflammation

In a recent study, published in *Nature*, Nordmann et al. identified Janus kinase inhibitors (JAKi) as a new treatment option for life-threatening toxic epidermal necrolysis (TEN). They showed this through spatial proteomics on human skin samples combined with in vivo administration of JAKi in individuals with TEN, demonstrating rapid reepithelization and recovery of patients.^1^ As TEN has a high mortality rate (30–50%),^2^ this study offers a new avenue for targeted treatment.

Cutaneous adverse drug reactions (CADR) comprise a spectrum of mild to severe conditions in dermatology that are mediated by drug-induced T cell-mediated hypersensitivity. Mild CADR are usually self-limiting, and individuals exhibit a maculopapular rash. Severe CADRs include entities of which a subset is characterized by blistering eruptions (epidermal necrolysis). These exhibit fatal skin involvement in Stevens-Johnson syndrome (SJS) and TEN with widespread epidermal detachment. Another entity of severe CADR is drug reaction with eosinophilia and systemic symptoms (DRESS), which is characterized by diffuse maculopapular eruption, systemic organ inflammation, and hematological abnormalities.

SJS and TEN are defined by epidermal and mucosal detachment. TEN is considered when epidermal detachment exceeds 10–35% of the body surface area.^2^ Clinical trials with large patient numbers in TEN remain extremely challenging due to rapid disease evolution.^3^ Immediate supportive care in an intensive care setting is of major importance in the treatment of TEN.^3^ While it has been acknowledged that the reaction is caused by extensive keratinocyte (KC) cell death and a CD8^+^ T cell-driven response, the question of how to target the main drivers of cytotoxicity against the epidermis remained.

Multiomic approaches with single-cell resolution have revolutionized our understanding of skin diseases. Thus far, molecular signatures have mostly been captured through transcriptomic approaches, while single-cell resolution of proteomics has been difficult to perform in tissue samples.

**Fig. 1:**
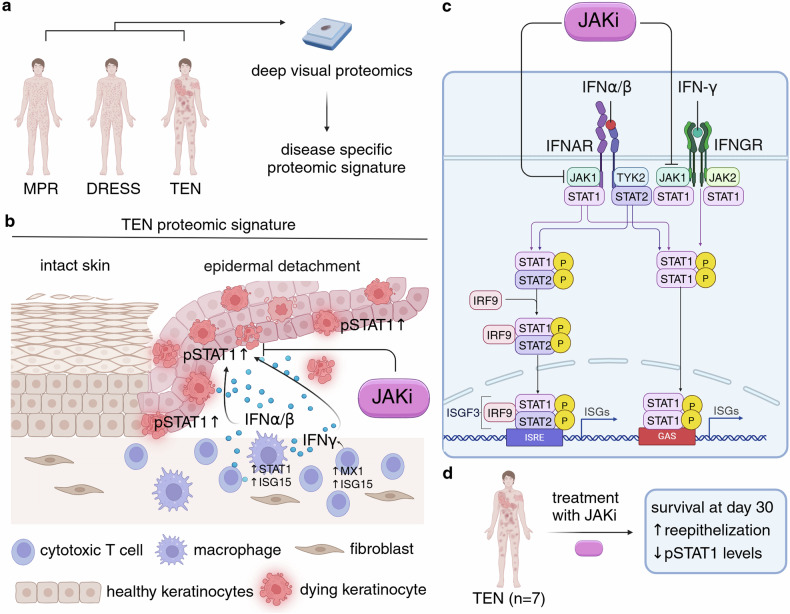
Spatial proteomics identifies JAKi as a new treatment option in TEN. **a** Experimental approach of spatial proteomics in maculopapular rash (MPR), drug eosinophilia with systemic symptoms (DRESS) and toxic epidermal necrolysis (TEN) using deep visual proteomics. **b** Summary of main findings in TEN by Nordmann et al. highlighting epidermal cell death and activation of STAT1 through type I and type II IFN signaling with infiltrating macrophages and T cells marked by high expression of IFN-induced genes. The cellular origin of type I/II IFN has not been investigated in this study. **c** Mechanism of JAKi in type I (IFNα/β) and type II (IFNγ) IFN signaling. **d** Treatment of TEN (*n* = 7) with JAKi and observed outcomes. Abbreviations: JAK Janus Kinase, JAKi JAK inhibitor, STAT Signal Transducer and Activator of Transcription, IFNAR Interferon alpha/beta Receptor, IFNGR Interferon gamma Receptor, IRF Interferon Regulatory Factor, ISGF3 Interferon-stimulated gene factor 3, ISRE Interferon Stimulation Response Element, ISG Interferon Stimulated Gene, GAS Gamma Interferon Activation Site. Created in BioRender. Kahlenberg, M. (2025) https://BioRender.com/a16k451

Nordmann et al. used deep visual proteomics (DVP) that combine imaging, artificial intelligence (AI)-based cell segmentation and laser dissection of individual cells followed by mass spectrometry-based proteomics to compare molecular signatures in self-resolving maculopapular rash with severe CADR (TEN, defined as >10% epidermal detachment and DRESS) (Fig. 1a). Notably, both KCs and immune cells in TEN exhibited a strong type I and type II Interferon (IFN) signature. The highest IFN score was present in macrophages, potentially through activation of IFNγ receptor signaling (Fig. 1b).^1^ In DRESS, proteomic changes were mainly driven by antigen presentation by KCs and a proliferative signature in immune cells. Comparison of proteomics in attached versus detached KCs identified inflammatory proteins such as MX1 and C3 as drivers of epidermal detachment in TEN.^1^ Importantly, together with targeted transcriptomics, pathway analysis revealed strong activation of the JAK-STAT pathway in TEN with high expression of IFNγ and activation of STAT proteins (Fig. 1b).^1^ Another study that investigated blister fluids from patients with TEN by single-cell RNA sequencing substantiated cellular crosstalk in TEN and showed that KCs are more vulnerable to CD8^+^ T cell-driven cell death: It identified HLA-class I-restricted antigen presentation by KCs and expansion of cytotoxic CD8^+^ T cells that derived from locally proliferating tissue-resident CD8^+^ T cells.^4^ It also showed that JAK-STAT signaling and TNF are upregulated in skin and immune cells of patients with TEN.^4^ The study of Nordmann et al. further corroborated the involvement of JAK-STAT signaling in TEN.

This study utilized AI-based annotation of cells, which comes with some limitations. These include potential exclusion of cells secondary to loss of morphology or cell death. This could apply to neutrophils undergoing neutrophil-extracellular trap (NET) formation. NETs were recently shown to promote KC necroptosis in SJS/TEN and might represent a target for therapy in CADR.^5^

JAKs are composed of four different proteins: JAK1, JAK2, JAK3, and Tyk2, which can be targeted through different JAKi with various specificity (Fig. 1c). In dermatology, JAKis has been recognized as an effective treatment option in multiple dermatologic conditions such as alopecia areata, atopic dermatitis, and vitiligo. Using a new autologous co-cell culture of PBMCs from TEN survivors and healthy KCs, JAKi efficiently blocked TEN survivor-derived PBMC-driven KC cell death (Fig. 1b).^1^ This finding led the authors to demonstrate the involvement of JAK-STAT signaling in two mouse models of TEN: In their first model, subcutaneous injection of smac (second mitochondria-derived activator of caspases) mimetics resulted in cutaneous inflammation and epidermal detachment, as seen in TEN. Administration of JAKi using tofacitinib (pan-JAKi), baricitinib (JAK1/2i) and, more specifically, abrocitinib and upadacitinib (JAK1i) resulted in remarkably reduced disease severity, reduction of cutaneous pSTAT1, cell death and faster reepithelization (Fig. 1b). A second humanized mouse model using PBMCs from TEN-survivors injected into immunodeficient mice also responded well to JAKi treatment, reflected by reduced ocular conjunctivitis and cell death.^1^

Finally, seven patients treated with TEN were treated off-label with JAKi (Fig. 1d). Strikingly, all seven patients survived 30 days with no side effects. One patient with a predicted mortality of 58.3% using a severity-of-illness score (SCORTEN) exhibited progressive disease despite high-dose intravenous steroids. JAKi stopped progression and led to signs of reepithelization four days after administration.^1^ Of note, SCORTEN has been shown to overestimate mortality in patients with TEN. Three of seven patients had received PD1-inhibitors for cancer and SCORTEN has not been validated in this population. Despite this comparatively small number of patients treated with JAKi, this study is groundbreaking and may provide a new path forward for this lethal disease. JAKis have a wide range of different activities and pharmacological properties. It remains to be determined which JAKi has the best efficacy in TEN. Given severe mucositis in TEN, ideal drug administration (oral/intravenous/subcutaneous) of different treatments should be assessed.

Common culprits of TEN are allopurinol, antibacterial sulfonamides, and aromatic antiepileptic medications.^2^ Predisposition to the development of CADR is due to certain HLA alleles.^2^ Of special interest will be whether certain subtypes of TEN respond better to JAKi than others. Specifically, non-drug associated TEN, which accounts for up to 25% of cases,^2^ remains to be tested for JAKi. Future clinical trials and head-to-head comparisons of different treatments in TEN, including systemic steroids, cyclosporin, intravenous immunoglobulins, TNF-inhibitors and JAKi will further elucidate the efficacy and safety of JAKi. STAT1 seems to be a crucial potentiator of inflammatory responses in TEN and it will be interesting whether specific STAT1-inhibition might be another option for patients. Combination therapy might be more effective than individual therapy. Another unmet need in TEN is the rapid availability of medication as patients often present too late for any treatment to benefit from. Ultimately, targeted treatment in TEN should be easily accessible, short-acting and be optimized by standardized supportive care.
